# 

**Published:** 1891-01

**Authors:** 


					﻿J/LKTTJJkRY, 1891-
OLD SERIBB
VoL XXV
<7 1855 <
NEW SERieS
Vol VII.—No. 11/
1HHK
jkMm
MNAl
UH. V. M. MILLER, M.D., LL.D.
VIRGIL 0. HARDON, M.D.
F. W. McRAE, M.D., M’n’g’ng Ed
f JAMES RHARRISON&Cffl
SUBSCRIPTION! 12.00 A YEAR.
				

